# The Repetitive Detection of Toluene with Bioluminescence Bioreporter *Pseudomonas putida* TVA8 Encapsulated in Silica Hydrogel on an Optical Fiber

**DOI:** 10.3390/ma9060467

**Published:** 2016-06-15

**Authors:** Gabriela Kuncová, Takayuki Ishizaki, Andrey Solovyev, Josef Trögl, Steven Ripp

**Affiliations:** 1 Institute of Chemical Process Fundamentals of the CAS, v.v.i., Rozvojová 135, 16500 Praha 6, Czech Republic; kuncova@icpf.cas.cz (G.K.); Ishizaki@seznam.cz (T.I.); solovyev@icpf.cas.cz (A.S.); 2 Faculty of Environment, Jan Evangelista Purkyně University in Ústí nad Labem, Králova Výšina 3132/7, 40096 Ústí nad Labem, Czech Republic; 3 Center for Environmental Biotechnology, The University of Tennessee, 676 Dabney Hall, Knoxville, TN 37996, USA; saripp@utk.edu

**Keywords:** bioluminescent biosensor, silica gel, encapsulation, optical fiber biosensor, whole cell bioreporter

## Abstract

Living cells of the *lux*-based bioluminescent bioreporter *Pseudomonas putida* TVA8 were encapsulated in a silica hydrogel attached to the distal wider end of a tapered quartz fiber. Bioluminescence of immobilized cells was induced with toluene at high (26.5 mg/L) and low (5.3 mg/L) concentrations. Initial bioluminescence maxima were achieved after >12 h. One week after immobilization, a biofilm-like layer of cells had formed on the surface of the silica gel. This resulted in shorter response times and more intensive bioluminescence maxima that appeared as rapidly as 2 h after toluene induction. Considerable second bioluminescence maxima were observed after inductions with 26.5 mg toluene/L. The second and third week after immobilization the biosensor repetitively and semiquantitatively detected toluene in buffered medium. Due to silica gel dissolution and biofilm detachment, the bioluminescent signal was decreasing 20–32 days after immobilization and completely extinguished after 32 days. The reproducible formation of a surface cell layer on the wider end of the tapered optical fiber can be translated to various whole cell bioluminescent biosensor devices and may serve as a platform for *in-situ* sensors.

## 1. Introduction

The integration of the *lux* gene cassette into microbial cells has created whole cell living bioreporters capable of sensing and responding to specific chemical, biological, and physical targets via the emission of bioluminescent light [1,2,3]. In soil and water, living whole-cell bioreporters can provide fast detection of potential threats that can then be characterized more fully by other analytical methods. In contrast to chromatographic analyses, bioreporters sense only bioavailable pollutants [4], and their application as environmental sensors has been previously reported in [5,6]. Although well-tested in laboratory-based whole-cell bioassays, examples of their interfacing with transducer elements to form deployable biosensors are less common [7,8,9,10,11,12].

The operational capabilities of devices with immobilized microorganisms are critically dependent upon the ability to maintain immobilized bioreporter populations in a viable state within a matrix that is strong enough to endure the rigors of the outside environment. The techniques for the immobilization of bioluminescent bioreporters that have been used or have a potential for application in the design of optical biosensors have been reviewed [13,14,15]. They comprise a broad spectrum of methods that include bacterial biofilms in a flow-through microreactor [16], physical attachment enhanced by the modification of a substrate or live cell’s surface [17], entrapment/encapsulation into natural or synthetic polymers [18], a combination of hydrogel entrapment and cryopreservation, plasma-deposited films, the application of photolithography, electrospinning, and electrodeposition [1,7,19]. Silica-based polymers possess some of the most desirable properties for immobilization of bioreporters, including biocompatibility, transparency, and chemical, thermal, and dimensional stability [20]. A previous study demonstrated that the bioluminescent bioreporter *Pseudomonas fluorescens* HK44 could be entrapped in a silica gel and remain viable for repetitive bioluminescence induction over several months [19].

The first bioluminescent bioreporters to be fixed on to optical fiber tips were entrapped in alginate [21,22]. Alginate gel containing living *lux*-expressing *Escherichia coli* bioreporters was applied on the fiber tip in a length of 1 cm. An optimal response to a model genotoxicant was achieved with six alginate/bacterial layers on a 1-cm exposed fiber-optic core [23]. To avoid irreversible analyte adsorption in the polymer/gel matrix and a prolonged response time, Premkumar *et al.* [24] embedded antibodies in a glutaraldehyde matrix and then attached *E. coli* bioreporter cells to the antibodies. Another approach to the fixation of bioluminescent reporter cells on the fiber end is the conjugation of biotinylated alginate microspheres with encapsulated cells to the surface of a streptavidin-coated optical fiber [25]. Polyak *et al.* [22] showed that, if the core diameter of the fiber was etched down, photon detection efficiency increased, although to a lesser extent than that expected from theoretical calculations. Immobilization of bioreporter cells on the wider end of a fiber taper improved the photon detection efficiency via an increase in the number of light sources [10,11].

*Pseudomonas putida* TVA8 [26] is a *tod-luxCDABE* bioluminescent bioreporter responding to the presence of benzene, toluene, ethylbenzene, and xylene (BTEX) compounds by the production of visible light. We have demonstrated operational conditions and selectivity of free *P. putida* TVA8 cells employed as a semiquantitative detector of water pollution [27]. *P. putida* TVA8 cells were further reproducibly encapsulated in silica gel adhered on the polished end of a quartz optical fiber. Core diameters of such optical fibers approach 600 μm, which limits the number of encapsulated cells that it can accommodate and consequently decreases biosensor sensitivity. This obstacle may be overcome by encapsulation of bioreporter cells on the wider end of a tapered optical fiber rather than the narrower, smaller-diameter opposing end [12]. We have demonstrated proof-of-concept for such a fiber optic biosensor arrangement using physical adsorption methods that were complex and deleterious to longer-term cell survival [9]. In this study, we explored a simpler and faster approach that bypassed physical adsorption requirements to create a biosensor for toluene using *P. putida* TVA8 encapsulated in silica gel attached to the wider end of a tapered optical fiber.

## 2. Materials and Methods

### 2.1. Chemicals and Solutions

All compounds were commercial products: hydrochloric acid, sodium chloride, and sodium hydroxide (Lach-Ner, Neratovice, Czech Republic), sodium and potassium phosphates, (Penta, Praha, Czech Republic), tryptone (Oxoid, Basingstoke, UK), yeast extract, kanamycin, and tetramethoxysilane (TMOS) (Sigma-Aldrich, St. Louis, MO, USA).

Phosphate buffered saline (PBS) (pH 7.4) was prepared by dilution from a previously prepared concentrated solution (×10) of PBS that contained KH_2_PO_4_ (17 mmol/L), Na_2_HPO_4_ (52 mmol/L), and NaCl (1.5 mol/L). Luria–Bertani media (LB) contained tryptone (10 g/L), yeast extract (5 g/L), and NaCl (10 g/L), pH 7.2 [28]. The LB + kan_50_ medium was prepared by the addition of a stock kanamycin solution (10 g/L) to a final concentration of 50 mg/L after autoclaving. Minimal salts medium (MSM) contained MgSO_4_·7H_2_O (0.1 g/L), NH_4_NO_3_ (0.2 g/L), trace elements (0.1 mL), 0.5 M phosphate buffer, pH 7.2 (100 mL), and distilled water (900 mL) [27].

### 2.2. Microorganism and Cultivation

*P. putida* TVA8 contains a chromosomally integrated *tod-luxCDABE* reporter gene cassette that enables bioluminescent gene expression to occur in the presence of toluene [26]. *P. putida* TVA8 cells were cultivated overnight in LB + kan_50_ medium with shaking at 28 °C to an optical density OD_600_ = 0.35. Cultures were washed once with an equal volume of MSM and then resuspended in an equal volume of MSM mixed with LB medium in a 3:7 ratio.

### 2.3. Entrapment of Cells into Silica Gel

Cell encapsulation was performed according to a procedure used in previous studies [19,29,30,31]. Briefly, tetramethoxysilane (TMOS, 4.1 g) was stirred with distilled water (2 mL), cooled for 5 min at 4 °C, and 0.1 M HCl (0.5 mL) was then slowly added. After disappearance of the two phases, a clear solution remained that was left to pre-polymerize for 24 h at 4 °C. The pre-polymerized TMOS (50 μL) was then mixed with 0.05 mol/L NaOH (50 μL) and the cell suspension (50 μL). This mixture, containing *P. putida* TVA8 at approximately 10^8^ cfu/mL, was individually dipped on the polished wider end face of tapered fiber, diameter_min_ = 0.6 mm, diameter_max_ = 10.6 mm, length = 22.5 cm, that was formed in the initial stage of drawing of polymer cladded silica fibers from a rod of Suprasil^®^ (kindly donated by the Institute of Photonics and Electronics ASCR, Prague, Czech Republic). Immediately after gelation, the quartz fiber end containing the *P. putida* TVA8 cells encapsulated in the silica gel drop was immersed in PBS. 

### 2.4. Scanning Electron Microscopy

For preparatin for scanning electron microscopy, the end of the fiber was left to dry under ambient conditions in a dry atmosphere for 2 days and then coated with gold using an EMITECH Sputter Coater K500X (Quorum Technologies Ltd. Laughton, East Sussech, UK) for 2 min under a sputtering current of 50 mA. Gold-coated samples were scanned with a Vega 3 Tescan scanning electron microscopy (SEM) (TESCAN Brno, s.r.o., Brno, Czech Republic). 

### 2.5. Toluene Induction and Bioluminescence Measurements

**Figure 1:**
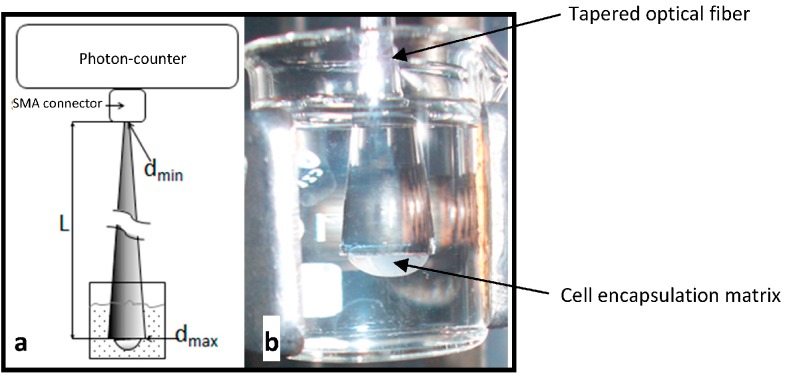
(**a**) The tapered optical fiber element contained at its wider distal end *P. putida* TVA8 cells encapsulated in a silica gel matrix. Immersion of the optical fiber into a solution of toluene instigated a bioluminescent response from the *P. putida* TVA8 cells that could be measured by an attached photon-counter module (L, length; d, diameter); (**b**) Photograph of the immersed optical fiber element 10 days after *P. putida* TVA8 immobilization.

The narrow end of the tapered optical fiber element was attached to a photon-counter detector (Perkin-Elmer 3954-P-087) (Perkin Elmer, Waltham, MA, USA) through an SMA optical fiber bare connector as depicted in Figure 1a. Bioluminescence from *P. putida* TVA8 was induced daily by immersion of the wider optical fiber end into a solution of toluene (26.5 or 5.3 mg/L in PBS) at ambient temperature (Figure 1b). The first induction was initiated 2 h after gelation with additional inductions occurring over a 34-day period. Before each induction, the fiber end with gel was washed with PBS. The bioluminescence intensity measured in counts per second (cps) was recorded every 10 s. The experiment, the encapsulation in the silica gel drop and induction with toluene (26.5 mg/L), was repeated twice using the same fiber. 

## 3. Results and Discussion

**Figure 2:**
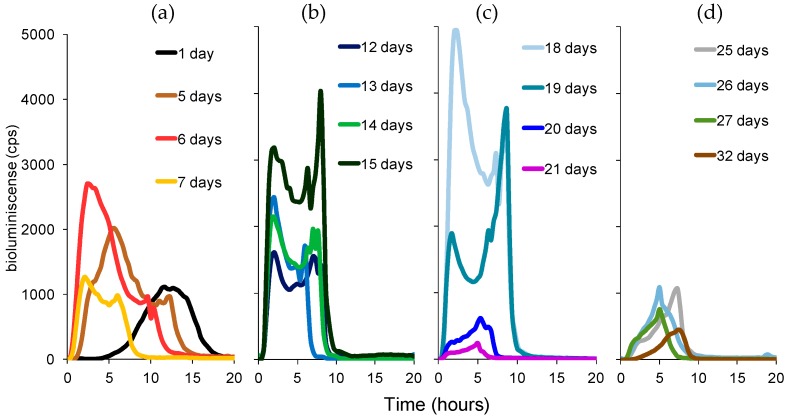
Representative bioluminescent emission profiles recorded from *P. putida* TVA8 bioreporter cells immobilized on the optical fiber after consecutive daily exposures to toluene at 26.5 mg/L over a total of 32 days (cps; counts/s). (**a**) days 1–9; (**b**) days 10–16; (**c**) days 17–24; (**d**) days 25–32.

**Figure 3:**
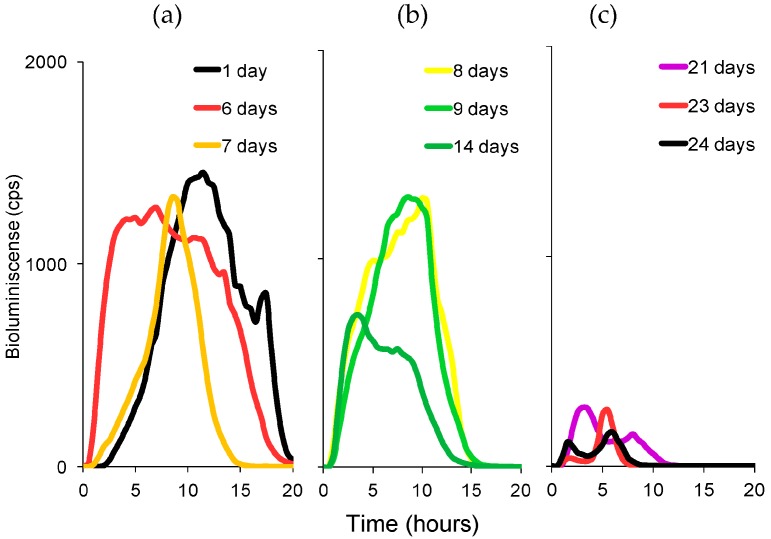
Representative bioluminescent emission profiles recorded from *P. putida* TVA8 bioreporter cells encapsulated in silica hydrogel on the optical fiber after consecutive daily exposures to toluene at 5.3 mg/L over a total of 24 days (fiber 1) (cps; counts/s). (**a**) days 1–7; (**b**) days 8–14; (**c**) days 15–24.

Representative bioluminescent emission profiles of *P. putida* TVA8 after daily inductions with toluene at 26.5 mg/L are depicted in Figure 2. The profiles are divided into four graphs spanning the entire 32-day exposure period. The sequence illustrates the initial low-level bioluminescent response (Figure 2a) as the bioreporter cell layer begins to form on the surface of the silica drop, which gradually increases in intensity over the following 1.5 weeks (Figure 2b,c) as the reporter cells grow and become stabilized on the silica matrix, and then declines (Figure 2d) as the reporter cells lose viability and begin to slough off of the silica drop concurrent with the drop’s dissolution on the fiber tip. Figure 3 shows the bioluminescent response profile for the end immobilized *P. putida* TVA8 after daily exposure to toluene at 5.3 mg/L. Under this toluene exposure scenario, bioreporter cells yielded predictably lower bioluminescent emission responses coincident with the lower level toluene induction that similarly decreased in intensity over this experiment’s 24-day exposure period.

**Figure 4:**
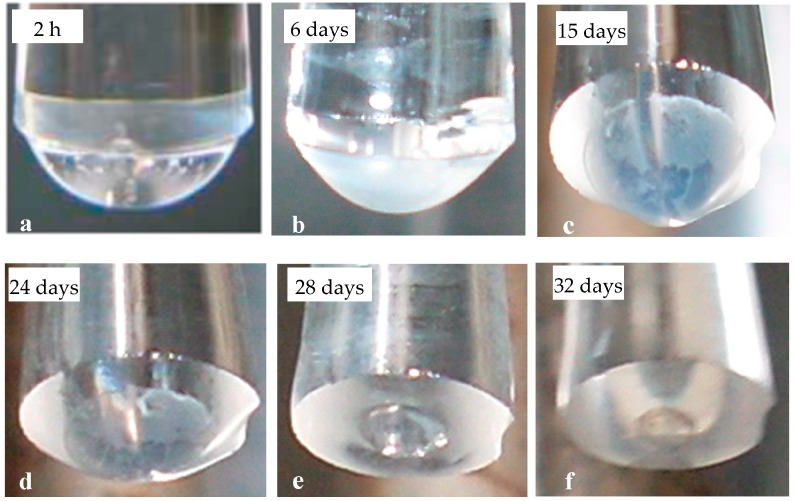
The fiber end with the silica hydrogel matrix (**a**) 2 h; (**b**) 6 days; (**c**) 15 days; (**d**) 24 days; (**e**) 28 days; and (**f**) 32 days after *P. putida* TVA8 immobilization and daily induction with toluene 26.5 mg/L. (portions of this figure were included in a recent review by the authors [12]).

Freshly immobilized *P. putida* TVA8 cells produced bioluminescence with a maximum occurring within 12 h regardless of whether they were induced with 26.5 mg/L (Figure 2a) or 5.3 mg/L toluene (Figure 3a). In the days thereafter, intensities of bioluminescence increased and times needed to reach maxima were shortened to 2 h (Figure 2b,c and Figure 3b). These changes in bioluminescent response profiles correspond to a change from a homogenous distribution of cells (Figure 4a), both inside and on the surface of the silica gel, to the cell free interior and surface covered with a dense cell population (Figure 4b). The increased density of the bioreporter cell population can be visualized in Figure 4a,b as an increase in opacity during the first week of testing. The silica gel matrix becomes condensed due to aging (restructuring after gelation). Therefore, inside the silica gel mass transport, the supply of oxygen and nutrients is limited, and cells preferably colonize the surface, leaving the center of the silica gel spot empty. Cell disappearance from the interior of the silica gel matrix has been observed in previous studies [32,33]. Figure 4 shows the dissolution of the silica gel over time, with the dimensions of the silica gel spot decreasing and leaving a gradually broadening fiber optic rim.

**Figure 5:**
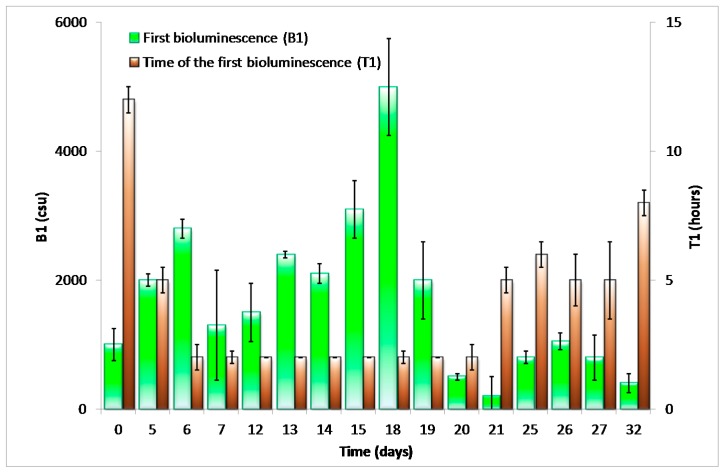
Variability of intensities of the first bioluminescence maxima (B1) and times of their appearance (T1) of the optical fiber elements with *P. putida* TVA8 encapsulated in silica hydrogel induced repeatedly with toluene (26.5 mg/L). Average ± standard deviations of two reproduced experiments are shown. The same tapered optical element was used in both experiments.

Over the following two weeks (Figure 2b,c), the first bioluminescence maxima and the times of their appearance (T1 and B1 in accordance with [9], Figure 5) remained stable. A second maxima appeared under higher (26.5 mg/L) concentrations of toluene exposure. We attribute this second peak to liberation and proliferation of free *P. putida* TVA8 cells. In a previous study [9] with TVA8 cells attached directly to an optical fiber, such second maxima were also observed, so they are not related to silica gel encapsulation. Specific bioluminescence of TVA8 cells increased with time and significantly dropped at the transition of the culture into stationary phase [26]. This transition was usually observed just after 10 h of growth [26,27], corresponding to observed maxima. At lower toluene concentration (5.3 mg/L), the bioluminescence maxima were correspondingly lower (Figure 3), and B1 were attained after 4 h instead of the 2 h needed at the concentration of 26.5 mg/L. The silica gel surface was covered with a thick cell layer, which was partially falling off by the 3rd week (Figure 4c,d).

**Figure 6:**
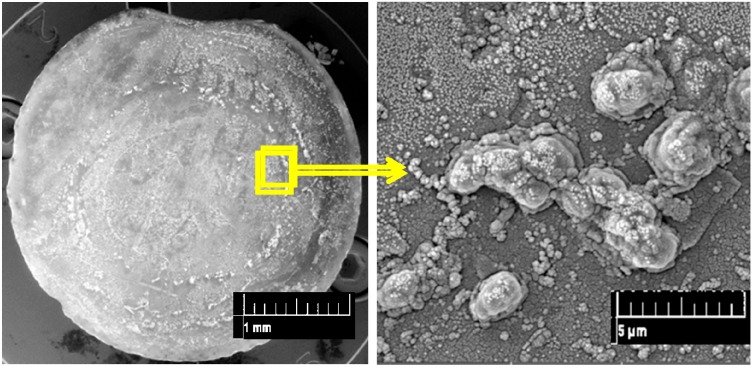
SEM micrograph of the wider end of the optic fiber showing *P. putida* TVA8 colonization after 34 days of repeated inductions.

In the last period, cell detachment and the decrement of the silica gel spot (Figure 4d,e) resulted in very low intensities of induced bioluminescence (Figure 2d). After 34 days, the gel spot completely disappeared, and only a few cells were observed on the end of the fiber (Figure 6). *P. putida* TVA8 did not colonize the pure quartz surface [9].

Between the 7th and 18th days, the measured intensities of bioluminescence were sufficiently high, and T1 remained stable (Figure 5). Within this period, the sensor functioned as a reliable and repetitive detector of toluene. Regardless of irregular intervals among inductions—24 h or one week—bioluminescence intensities corresponded roughly to the toluene concentrations (intensities of bioluminescence maxima induced with 26.5 mg/L toluene were always at least double as compared to those with 5.3 mg/L toluene).

In order to assess the variability of the response, a repetition of the experiment (immobilization and bioluminescence inductions) using the same tapered optical element was carried out (Figure 5). All repetitions (also with various tapered elements and thickness of silica gel) showed the same changes of bioluminescence intensities and response times that corresponded to the surface cell colonization: (1) the development of a cell layer on the surface; (2) a relatively stable cell layer on the surface; (3) cell detachments. A period of cell layer stability was decreased due to diminishment of the silica gel spot.

A biofilm-like layer from the bioreporter cells, attached directly on the surface of the wider end of the optical fiber, should have had a longer lifespan, but the biofilm-like layer unpredictably waned within repetitive inductions. In addition, the microorganisms slowly overgrow the fiber end (3–5 days) [9]. The method of biosensor preparation, described here, exploits encapsulation into silica gel to reproducibly create a surface cell layer. Silicic acid species releasing from the silica gel might fortify this cell layer in a similar manner as described in [17]. Fluctuations of intensities of bioluminescence maxima were related to biofilm-like layer formation (compare Figure 4b,c—day 6 and day 15). The highest bioluminescence maxima were produced by compact biofilm-like layers (days 6 and 7). A fall out of a part of this layer (see spots on Figure 4c) caused a decrease of bioluminescence maxima. Re-overgrowth of the silica gel surface with cells resulted in restoration of high bioluminescence maxima. Times of biofilm-like layer formation were reproducible.

In a previous study [9], *P. putida* TVA8 was immobilized on an optical fiber by physical adsorption of the cells to the fiber’s wider end, which had been modified with 3-aminopropyltriethoxysilane. This biosensor was induced with toluene at 26.5 mg/L 68 times over 135 days. The intensities of induced bioluminescence varied according to changes in the number and viability of the reporter cells in the biofilm-like layer. The main disadvantages of this biosensor configuration were the length of time needed for preparation of the cell layer and continual changes of analytical responses. In this work, we applied an optimized route toward [19,29,30,34] silica-gel-encapsulation of living bioreporters on to optical fibers. In contrast to physical adsorption, this technique enables reproducible fixation of a desired amount of cells on the top of the optical fiber within an hour [35]. As a result, the stability of the bioluminescence responses was improved, but only for a 10-day period, which is comparable to another whole cell biosensor of polychlorinated biphenyls (two weeks’ stability) [34] or a biosensor of L-asparagine (40 days storage stability) [36].

The major obstacle of *in*-*situ* analytical applications of bioreporters is their genetically-modified-organism (GMO) nature. Strict legislation in the EU, the USA, and other developed countries practically bans field applications. Nevertheless, the number of laboratories possessing the contained-use permission is increasing because novel analytical tests, such as genotoxicity [37] and endocrine disruption [38] assays, use GMOs. The biosensor described here is conceivable as a probe (like a pH electrode) for the inexpensive detection of toluene and other BTEX contaminants in water samples, and provides a facile means for transference of bioluminescent light signals from remote and inhospitable environments to a measurement interface. Screening with this biosensor can supplement established chromatographic analysis with added information regarding BTEX bioavailability.

## 4. Conclusions

A long-term response of an optical biosensor utilizing the bioluminescent bioreporter *P. putida* TVA8 encapsulated via a sol-gel route on the tip of a tapered optical fiber was tested. A slow shift of the bacterial population from the bulk gel to its surface, a formation of biofilm, and its detachment and gel dissolution was observed. Consequently, the achieved bioluminescence maxima and the times of their appearance were affected with up to nearly complete extinction after 30 days. These reproducible changes, however, resulted in sufficient and stable bioluminescence responses between the 7th and 18th day and enabled repeatable toluene detection in this period. More robust testing under real-world environmental contamination scenarios will be critical toward the transitioning of this biosensor beyond its proof-of-concept stage.

## Abbreviations

The following abbreviations are used in this manuscript:

B1: intensity of the first bioluminescence maxima (in accordance to [9], Figure 5)
BTEX: benzene, toluene, ethylbenzene, and xylene
OD_600_: optical density determined at λ = 600 nm
SEM: scanning electron microscopy
SMA connector: sub miniature version A optical fiber connector
T1: time of appearance of the first bioluminescence maxima (in accordance to [9], Figure 5)
